# Research on Integrated Modularization of Supercritical Carbon Dioxide System for Aircraft Carrier Nuclear Power

**DOI:** 10.3390/e27111154

**Published:** 2025-11-14

**Authors:** Shengya Hou, Junren Chen, Fengyuan Zhang, Qiguo Yang

**Affiliations:** 1 Institute of Power Machinery and Engineering, School of Energy and Power Engineering, University of Shanghai for Science and Technology, Shanghai 200240, China; 2 Department of Chemical Engineering, University College London, Torrington Place, London WC1E 7JE, UK

**Keywords:** aircraft carrier, nuclear power, supercritical carbon dioxide cycle, integration comparative analysis

## Abstract

This paper innovatively presents an integrated nuclear-powered supercritical carbon dioxide (S-CO_2_) system for aircraft carriers, replacing the conventional secondary-loop steam Rankine cycle with a regenerative S-CO_2_ power cycle. The system comprises two modules: a nuclear reactor module and a S-CO_2_ power module. Comprehensive thermodynamic, economic, and compactness analyses were conducted, using exergy efficiency, levelized energy cost (LEC), and heat transfer area per unit power output (APR) as objective functions for optimization. Parameter analysis revealed the influence of key operating parameters on system performance, and a multi-objective optimization approach based on genetic algorithms was employed to determine optimal system parameters. The results indicate that the system achieves an exergy efficiency of 45%, an APR of 0.168 m^2^ kW^−1^, and an LEC of 2.1 cents/(kW·h). This high compactness, combined with superior thermodynamic and economic performance, underscores the feasibility of the S-CO_2_ system for integration into nuclear-powered aircraft carriers, offering significant potential to enhance their overall performance and operational efficiency.

## 1. Introduction

As a cornerstone of modern naval power, the aircraft carrier serves not only as a mobile platform for maritime operations but also as a symbol of national sovereignty and strategic intent. In the complex international environment of the 21st century, the strategic importance of aircraft carriers has grown increasingly significant. With formidable strike capabilities, these vessels are also capable of performing a wide range of non-traditional security missions, including humanitarian assistance and disaster relief. Therefore, improving the performance and operational efficiency of aircraft carriers is crucial for safeguarding national security and promoting broader global interests [1]. The commissioning of the world’s first nuclear-powered aircraft carrier, the USS Enterprise, marked a transformative shift in global carrier operations [2]. Nuclear propulsion removed traditional limitations on speed and endurance, enabling rapid deployment and enhanced tactical flexibility. By significantly reducing the need for large quantities of onboard fuel storage, nuclear-powered carriers were able to reallocate interior space for additional armaments, aviation fuel, logistical supplies, or improved crew living conditions. Furthermore, decreased dependence on oil supply convoys streamlined fleet logistics, improved safety standards, and enhanced the overall operational flexibility and effectiveness of carrier battle groups [3].

At present, the power conversion cycle utilized in nuclear-powered aircraft carriers predominantly relies on the steam Rankine cycle. As a conventional propulsion technology, the steam Rankine cycle system provides substantial power output and proven reliability. Nevertheless, it is associated with several notable disadvantages, such as a complex system configuration, considerable spatial demands, and prolonged startup times [4,5]. The equipment required for the steam Rankine cycle is characterized by its large size and complex design, resulting in considerable spatial requirements and increased maintenance costs. These factors constrain the efficient use of internal space within the vessel [6]. Furthermore, critical components such as steam generators demand strict material specifications, and extended operation inevitably leads to corrosion, which undermines system stability [7,8]. Steam oxidation frequently results in operational issues, including oxide scale peeling, pipe blockages, and pipe ruptures [9]. Consequently, nuclear-powered carriers often require scheduled port visits for maintenance, limiting their ability to maintain continuous full-capacity operations.

With advancements in energy conversion technologies, the supercritical carbon dioxide (S-CO_2_) Brayton cycle has attracted growing interest as a promising alternative to conventional steam cycles. The theoretical basis for utilizing carbon dioxide as a working fluid in power generation dates back to the late 1940s, when Sulzer Brothers patented a Brayton cycle incorporating partial condensation of CO_2_ [10]. The system’s adaptability to diverse heat sources stems from the relatively accessible critical conditions of CO_2_—31 °C and 7.38 MPa [11,12,13]. In proximity to its critical point, CO_2_ exhibits high density and low viscosity, combining the favorable properties of both liquids and gases. These characteristics contribute to reduced compressor power consumption and diminished flow-induced friction losses. Moreover, the chemically stable and non-corrosive nature of CO_2_ enhances overall system safety and reliability [14].

The S-CO_2_ Brayton cycle is distinguished by its compact design, requiring substantially less space and weight in comparison to conventional steam cycles [15,16]. By operating entirely in the supercritical state, the cycle facilitates highly efficient heat transfer, thereby enhancing overall energy conversion efficiency [17]. Xu et al. conducted a comparative analysis between the S-CO_2_ Brayton cycle and the steam Rankine cycle, reporting a net power generation efficiency of 49.01% for the S-CO_2_ system, surpassing the 48.12% efficiency of the advanced Rankine system [18]. Likewise, Kizilkan et al. assessed the applicability of the S-CO_2_ Brayton cycle in waste heat recovery applications, achieving energy efficiencies of 27.6%, which outperformed the 24.2% efficiency of the conventional steam Rankine cycle [19]. Cheng et al. evaluated four different configurations of the S-CO_2_ Brayton cycle with respect to thermodynamic and techno-economic performance, employing the multi-criteria decision-making method. Considering factors such as safety, thermodynamic efficiency, technical economy, and structural compactness, their study identified a molten salt reactor integrated with a recompression cycle as the most suitable configuration for nuclear energy utilization [20].

For nuclear-powered aircraft carriers, the adoption of the S-CO_2_ Brayton cycle presents several notable advantages. The S-CO_2_ cycle typically operates with an expansion ratio of 2–4, and CO_2_ maintains a significantly higher density than steam even at elevated temperatures [21]. These characteristics position it as a promising alternative to the currently employed steam cycle in nuclear-powered aircraft carriers, thereby justifying further investigation. Through the design and comparative analysis of S-CO_2_ cycle power systems for nuclear-powered vessels, Li et al. determined that the partial cooling cycle represents the most suitable configuration. It offers high thermal efficiency, reduced system volume, lower operational costs, and relatively stable performance under partial load conditions [22].

Lee et al. investigated a system integrating a molten salt reactor with an S-CO_2_ Brayton cycle for application in nuclear-powered ships. Their sensitivity analysis revealed that the optimal cycle efficiency of 47.78% was achieved at a shunt ratio of 0.7 and a pressure ratio of 2.94. Compared to a conventional steam-based Rankine cycle reference design, the S-CO_2_ cycle demonstrated approximately 12% greater efficiency [23]. Similarly, Gumus et al. examined an S-CO_2_-RO (reverse osmosis) cogeneration system designed to simultaneously meet electricity and freshwater requirements during naval operations. Their findings indicated a thermal efficiency of 40.5%, an electrical output of 121.6 MW, and a daily freshwater production capacity of 1531.6 m^3^, achieved with only 0.12% (145.9 kW) of the generated power [24].

In summary, the research and potential implementation of the S-CO_2_ Brayton cycle in nuclear-powered aircraft carriers represent a significant technological advancement and a strategic initiative aimed at enhancing naval operational capabilities. However, previous studies have not sufficiently addressed the underlying mechanisms for adapting the S-CO_2_ power cycle to nuclear propulsion systems, nor have they provided comprehensive optimization of the integrated system architecture to determine the most appropriate cycle configuration and parametric settings. Therefore, this study seeks to elucidate the compactness mechanism of the S-CO_2_ power system by analyzing its thermophysical properties through comparative assessments. Furthermore, by conducting parametric analysis and applying multi-objective optimization techniques, the overall system performance can be effectively enhanced. The objective is to replace the conventional steam-based power generation system with S-CO_2_ power cycle, while maintaining the existing nuclear reactor of the aircraft carrier unchanged. This approach provides a critical foundation for the practical implementation of nuclear-powered S-CO_2_ power systems.

## 2. Cycle Description

**Figure 1:**
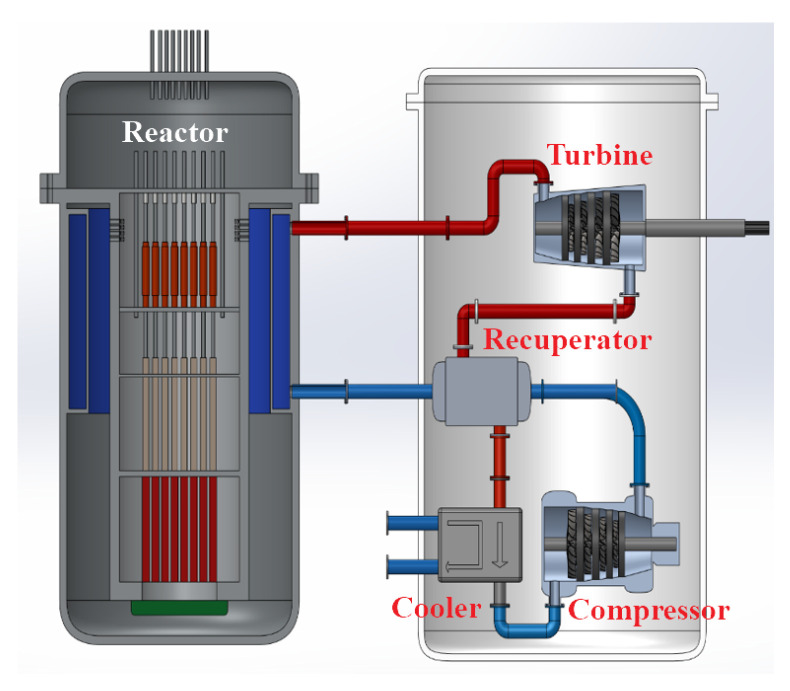
The 3D Schematic Illustration of the System.

**Figure 2:**
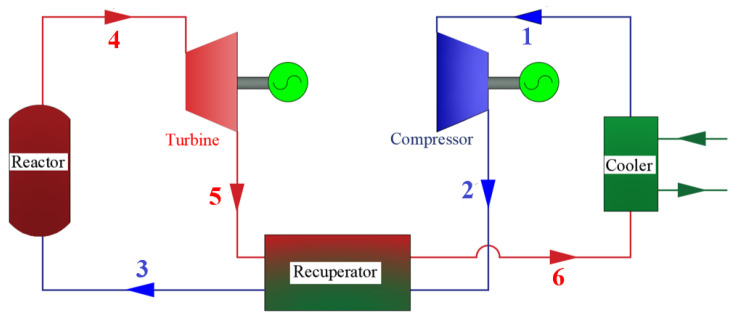
Schematic diagrams of the proposed systems.

This paper proposes a nuclear-powered S-CO_2_ integrated system for aircraft carriers, which replaces the conventional secondary steam Rankine cycle in the nuclear power plant with an S-CO_2_ power cycle. The system architecture is depicted in Figure 1, comprising two primary modules: the nuclear reactor module and the S-CO_2_ power module. Figure 2 presents schematic diagrams of the proposed systems. In the supercritical carbon dioxide power module, the red and blue lines denote the high-temperature fluid and the low-temperature fluid, respectively.

The nuclear reactor incorporates multiple safety mechanisms, including a robust reactor pressure vessel and redundant safety systems. It utilizes enriched uranium as fuel, with control rods regulating the reaction rate by absorbing excess neutrons. Each fission event releases significant thermal energy, which is transferred to high-pressure water serving as the coolant. Upon exiting the reactor core, the coolant’s temperature increases from 286.5 °C at the inlet to 319.5 °C at the outlet. High-pressure water is employed as the reactor coolant. The high-temperature coolant subsequently flows into the S-CO_2_ heat exchanger, transferring thermal energy to the S-CO_2_ working fluid. The commonly used cycle forms of supercritical carbon dioxide cycle are regenerative cycle and recompression cycle. Due to the medium and low temperature of the core, if the recompression cycle is adopted, the output power will not be significantly increased compared with the regenerative cycle, but the structure will be more complex. Therefore, under this heat source condition, the regenerative cycle is more suitable.

The high-temperature, high-pressure S-CO_2_ then enters the power module, driving a turbine mechanically coupled to the gearbox and propeller shaft, thereby propelling the vessel. After passing through the turbine, the S-CO_2_, still at a relatively high temperature, flows into the regenerator, where it transfers heat to the carbon dioxide exiting the compressor, enhancing the overall thermodynamic efficiency of the cycle. The cooled S-CO_2_ from the regenerator subsequently enters a seawater-cooled cooler before being directed into the compressor. Following compression, the high-pressure S-CO_2_ is pre-heated in the regenerator and returned to the heat absorber to complete one cycle. Detailed parameters of the nuclear reactor are provided in Table 1.

The assumptions of this research are as follows:

(1) The system maintains a stable operating state.
(2) The pressure and temperature of the environmental conditions are 1.013 bar and 25 °C, respectively.
(3) The variations in kinetic energy and potential energy can be negligible.

## 3. Mathematical Modeling

To comprehensively evaluate the performance of the cycle system, a set of integrated performance models is established, including thermodynamic analysis, exergy analysis, space utilization assessment, and exergoeconomic analysis. The selected objective functions include output power, thermal efficiency, exergy efficiency, levelized energy cost (LEC) per unit of power output, and heat exchanger area per unit of power output (APR). The selection of these objective functions is based on the following considerations. The primary purpose of the power cycle system is to generate electricity externally. Therefore, output power, which directly reflects the amount of electricity produced, is the most critical performance indicator and of central concern to system users. Thermal efficiency quantifies the proportion of input thermal energy that is converted into useful work, thereby reflecting the system’s energy conversion capability from a “quantity” perspective. In contrast, exergy efficiency evaluates system performance from a quality perspective by employing exergy balance analysis, which considers both the quantity and the quality of energy. This approach enables the identification of inefficiencies and exergy destruction within the system, providing insights for targeted improvements and optimization strategies. However, evaluating system performance solely based on energy output would be insufficient without considering economic implications. Hence, the LEC is introduced as an economic indicator to assess the investment and operational costs associated with each unit of electricity generated, supporting the evaluation and optimization of the system’s economic feasibility. Furthermore, in the context of nuclear-powered aircraft carriers, spatial constraints are of significant importance. To account for this, the APR is included as an objective function. A higher APR indicates greater spatial requirements, which may limit system integration and operability. By optimizing APR, the system’s compactness and overall layout efficiency can be improved, which is particularly crucial in applications with limited available space.

### 3.1. Thermodynamic Analysis Model

**Figure 3:**
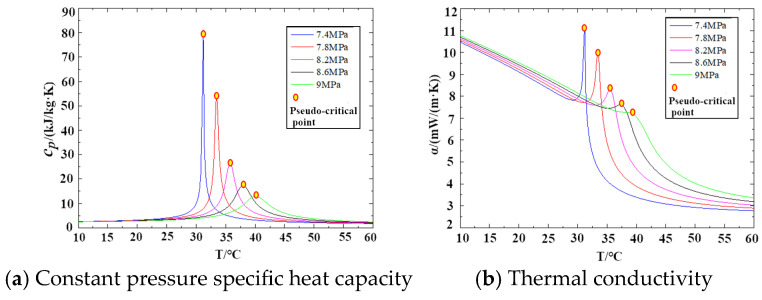

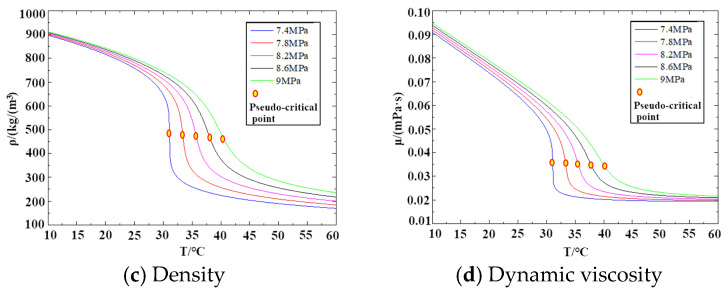
Changes in thermal properties near the critical zone of CO_2_ with temperature and pressure.

Thermodynamic analysis is based on the first law of thermodynamics, which states that the change in internal energy of a working fluid equals the sum of the heat added to the system and the work done on the system by the surroundings. In this study, output power and thermal efficiency are selected as the key objective functions for evaluating system performance. In this study, the required physical properties, such as density, dynamic viscosity, thermal conductivity, and specific heat capacity, are calculated by invoking the NIST physical property database. The variations in the physical properties of carbon dioxide with temperature and pressure are presented in Figure 3. It is important to note that in the vicinity of the critical point, the physical properties exhibit abrupt changes. Consequently, the heat transfer performance and pressure drop of the heat exchanger, as well as the performance of the compressor, demonstrate a high degree of sensitivity to the physical property parameters [25,26,27,28,29,30].

#### 3.1.1. Heat Exchanger Model

In the supercritical state, carbon dioxide exhibits gas-like behavior, leading to relatively inferior heat transfer characteristics. Printed circuit board heat exchangers (PCHEs), on the other hand, are capable of offering a large heat transfer area within a compact footprint, thereby achieving high heat transfer efficiency. Additionally, PCHEs demonstrate exceptional performance under high-pressure operating conditions. Therefore, in this study, the printed circuit board heat exchanger is identified as the preferred heat exchange device for the supercritical carbon dioxide cycle.

The heat balance equation for the heat exchanger can be expressed as:

(1) $$\dot{Q}={\dot{m}}_{h}(h_{hi}-h_{ho})={\dot{m}}_{c}(h_{co}-h_{ci})$$

where ${\dot{m}}_{h},{\dot{m}}_{c}$ are mass flow rate of hot fluid and cold fluid, kg/s. $h_{hi},h_{ho}$ account for the specific enthalpy values of the inlet and outlet of hot fluid, J/kg, and $h_{ci},h_{co}$ are specific enthalpy values of the inlet and outlet of cold fluid, J/kg.

The total amount of heat exchange can also be written as:

(2) $$\dot{Q}=UA\Delta T$$

where A denotes the heat transfer area on the supercritical carbon dioxide side, m^2^. In addition, the overall heat transfer coefficient (*U*) is given by

(3) $$U=\frac{1}{\frac{1}{h_{h}}+\frac{1}{h_{c}}\frac{A_{h}}{A_{c}}+R}$$

where $h_{h},h_{c}$ are heat transfer coefficients on the hot side and the cold side, W/(m^2^·K). $A_{h},A_{c}$ denote heat exchange area of the hot side and the cold side, m^2^, and R is tube wall thermal resistance, (m^2^·K)/W.

The logarithmic mean temperature difference is defined as:

(4) $$\Delta T_{m}=\frac{\Delta T^{\prime }-\Delta T^{\prime \prime }}{\ln\frac{\Delta T^{\prime }}{\Delta T^{\prime \prime }}}$$

(5) $$\Delta T^{\prime }=T_{hi}-T_{co}$$

(6) $$\Delta T^{\prime \prime }=T_{ho}-T_{ci}$$

where $T_{hi},T_{ho}$ are the inlet and outlet temperatures of hot fluid, and $T_{ci},T_{co}$ denote the inlet and outlet temperatures of cold fluid.

**Figure 4:**
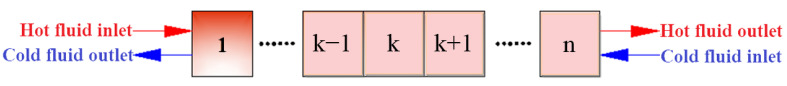
Distribution of heat exchanger units.

The logarithmic mean temperature difference is applicable under the condition where the thermophysical properties of both the hot and cold fluids remain constant. Nevertheless, in this study, there are significant differences in the thermophysical parameters of the hot and cold fluids. In particular, the specific heat at constant pressure experiences a sharp increase at the pseudo-critical point temperature. Thus, the overall heat transfer coefficient can be calculated using the following approach:

(1) Divide the printed circuit board heat exchanger into *n* units with equal heat exchange amounts, as depicted in Figure 4;
(2) Compute the average temperature difference between carbon dioxide and water within each unit, $T_{m,i}$, where *i* = 1, 2, …, *n*. The minimum heat-transfer temperature difference in the heat exchanger is 5 K.

Based on the above analysis, the following relations can be listed:

(7) $$\Delta Q_{k}=\frac{Q_{k}}{n}$$

(8) $$\Delta Q_{k}=U\Delta A_{k}\Delta T_{mk}$$

(9) $$\Delta Q_{k}={\dot{m}}_{h,k}(h_{hk.i}-h_{hk,o})={\dot{m}}_{c,k}(h_{ck,o}-h_{ck,i})$$

(10) $$\sum \Delta A_{k}=A$$

where $\Delta Q_{k},\Delta A_{k},\Delta T_{mk}$ are the heat exchange (W), heat exchange area (m^2^), and temperature difference (°C) of the *k*th unit, respectively.

The efficiency of each unit is:

(11) $$\varepsilon =\frac{\Delta Q_{k}}{\Delta Q_{k,\max}}$$

where $\Delta Q_{k,\max}$ is the ideal maximum heat transfer capacity of the *k*th unit, which is defined as:

(12) $$\Delta Q_{k,\max}=C_{k,\min}(T_{hk,i}-T_{ck,i})$$

In the equation, $C_{k,\min}$ represents the smaller heat capacity of the two fluids, namely:

(13) $$C_{k,\min}=min\left({\dot{m}}_{h}c_{Ph,k},{\dot{m}}_{c}c_{Pc,k}\right)$$

Consequently, the effectiveness of the heat exchanger unit can alternatively be defined as

(14) $$NTU_{k}=\frac{U\Delta A_{k}}{C_{k,\min}}$$

The efficiency of the heat exchanger unit can also be defined as:

(15) $$\varepsilon_{k}=\frac{1-\exp\left(-NTU_{k}\left(1-R_{k}\right)\right)}{1-R_{k}\exp\left(-NTU_{k}\left(1-R_{k}\right)\right)}$$

where $R_{k}$ denotes the ratio of the smaller heat capacity to the larger heat capacity of the two fluids, namely:

(16) $$R_{k}=\frac{C_{k,\min}}{\max\left({\dot{m}}_{h}c_{ph,k},{\dot{m}}_{c}c_{pc,k}\right)}$$

In the formula, *c_ph,k_* and *c_pc,k_* denote the specific heat capacities of the hot fluid and the cold fluid in the *k*th unit, respectively.

In the design procedure, the outlet temperature of the cold fluid is hypothesized and regarded as the iterative variable. The ε-NTU method is adopted to compute the outlet temperature of the hot fluid in the heat exchanger unit. Through iterative computations, the inlet temperature of the cold fluid is derived. Once the deviation between this value and the specified inlet temperature of the cold fluid lies within the predefined range, the iteration concludes [31,32].

The Reynolds number of the heat transfer unit is defined as follows:

(17) Rek=ρkvkdkμk=mkdkμkAk

In the formula, $\rho_{k},\mu_{k},v_{k}$ are the density of the fluid (kg/m^3^), dynamic viscosity (Pa·s), and flow velocity (m/s), respectively. $A_{k},d_{k}$ denote the area of the cross-section of the channel (m^2^) and the hydraulic diameter (m), respectively.

The Prant number of the heat exchange unit is defined as:

(18) $${\mathrm{Pr}}_{k}=\frac{c_{p,k}\mu_{k}}{k_{k}}$$

where $k_{k}$ is the thermal conductivity of fluid.

The pressure drop of the fluid in the heat exchanger unit is expressed as:

(19) $$\Delta P_{k}=f_{k}\frac{L_{k}m_{k}^{2}}{2d_{h,k}\rho_{k}A_{k}^{2}}$$

where $f_{k}$ denotes the friction factor of the fluid, and $L_{k}$ is the qualitative length of heat exchanger unit.

The Nussel number of supercritical carbon dioxide flowing in the tube is defined by the Gnielinski correlation as:

(20) Nu=(fk/8)(Re−1000)Pr1.07+(900/Re)−[0.63/(1+10Pr)]+12.7fk/8(Pr2/3−1)

where $f_{k}$ is Darcy friction factor, whose expression is:

(21) fk=1.82lnRe−1.64−2

**Figure 5:**
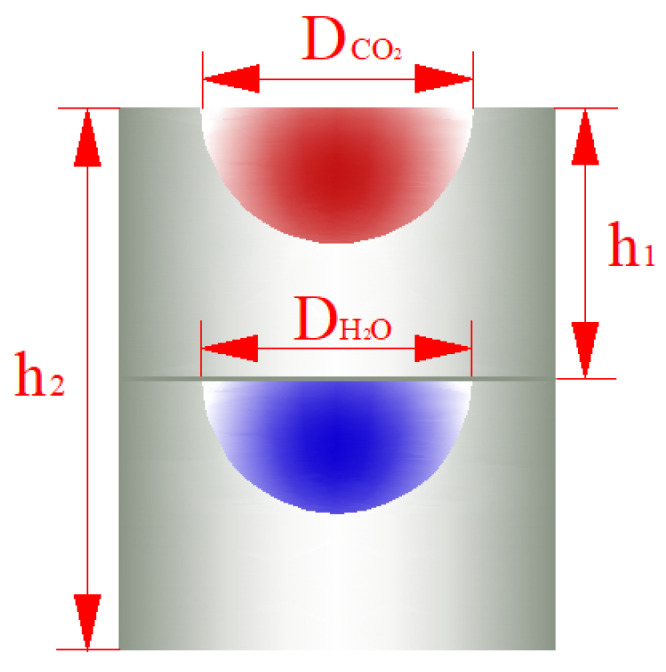
Internal structure diagram of printed circuit board heat exchanger.

In this study, the printed circuit board heat exchanger employed features a straight-through channel configuration. The cross-section of the channel is semicircular, as depicted in Figure 5. The red channels represent the hot side, while the blue channels represent the cold side. The cross-sectional diameter, plate thickness, and flow channel structure of the hot side are identical to those of the cold side channels.

#### 3.1.2. Turbomachinery Model

Supercritical carbon dioxide generates power output through a turbine expansion process. The supercritical carbon dioxide turbine plays a critical role in ensuring the safe, reliable, and efficient operation of the supercritical cycle system. Additionally, it serves as a key element that highlights the compact design and high energy density of this thermodynamic cycle.

Carbon dioxide undergoes an isentropic expansion process within the expander. Under ideal conditions, the entropy and specific enthalpy at the expander outlet can be determined as follows:

(22) $$s_{t,e^{\prime }}=s_{t,i}$$

(23) $$h_{t,e^{\prime }}=h(s_{t,e^{\prime },}P_{t,e})$$

where $s_{t,i}$ denotes the specific entropy of the fluid at the turbine inlet, J/kg·K; $P_{t,e}$ represents the pressure of the fluid at the turbine outlet, kPa.

The actual specific enthalpy of carbon dioxide at the turbine outlet is determined by:

(24) $$h_{t,e}=h_{t,i}-\left(h_{t,i}-h_{t,e^{\prime }}\right)\eta_{t}$$

where $h_{t,i}$ denotes the specific enthalpy at the turbine inlet, J/kg; $h_{t,e^{\prime }}$ represents the isentropic specific enthalpy at the turbine outlet, J/kg. $\eta_{t}$ signifies the isentropic efficiency of the turbine, which is determined by two key parameters: the specific speed and the specific diameter. The isentropic efficiency is represented by a function obtained from the Balje contour diagram specific to turbines.

(25) $$N_{s}=\frac{w\sqrt{V}}{\Delta {h_{s}}^{\frac{3}{4}}}$$

(26) $$D_{s}=\frac{D\Delta {h_{s}}^{\frac{1}{4}}}{\sqrt{V}}$$

where *w* denotes the angular velocity of the shaft; *V* represents the volumetric flow rate; *D* refers to the turbine diameter; and $\Delta h_{s}$ indicates the isentropic enthalpy change between the inlet and outlet.

The shaft power of the supercritical carbon dioxide turbine can be expressed as follows:

(27) $${\dot{W}}_{t}={\dot{m}}_{t}\left(h_{t,i}-h_{t,e}\right)$$

The cost rate of the supercritical carbon dioxide expander is mathematically expressed as follows:

(28) $$Z_{tur}=479.34m(\frac{1}{0.93-\eta_{tur}})\ln(PR_{tur})\cdot (1+\exp(0.036T_{1}-54.4))$$

(29) $$PR_{t}=\frac{P_{t,i}}{P_{t,e}}$$

where ${\dot{m}}_{t}$ denotes the mass flow rate of the working fluid at the turbine inlet; $\eta_{t^{\prime }}$ represents the ideal isentropic efficiency of the turbine; $PR_{t}$ signifies the pressure ratio across the turbine; $T_{t,i}$ refers to the inlet temperature of the working fluid; $P_{t,i},P_{t,e}$ denote the inlet and outlet pressures of the turbine; $\alpha_{1},\alpha_{2},K_{t}$ are coefficients that depends on the specific type and structural design of the expander.

The exergy loss of carbon dioxide within the turbine can be expressed as follows:

(30) $${\dot{E}}_{D,t}=\dot{m}(e_{t,i}-e_{t,e})-{\dot{W}}_{t}$$

where $e_{t,i}$ and $e_{t,e}$ represent the specific exergy values of carbon dioxide at the compressor inlet and outlet, respectively.

The net output power of the supercritical carbon dioxide cycle can be expressed as:

(31) $$\dot{W}={\dot{W}}_{t}-{\dot{W}}_{c}$$

where ${\dot{W}}_{t}$ represents output power of the carbon dioxide turbine; ${\dot{W}}_{c}$ denotes the power consumption of the compressor.

The thermal efficiency of the supercritical carbon dioxide cycle can be expressed as:

(32) $$\eta_{th}=\frac{\dot{W}}{\dot{Q}}$$

where $\dot{Q}$ denotes the rate of heat absorption in the system.

### 3.2. Exergy Analysis Evaluation Model

Exergy analysis is a thermodynamic methodology grounded in the second law of thermodynamics, used to evaluate the degree of thermodynamic perfection within an energy conversion system. Compared with thermal efficiency, exergy efficiency provides a more comprehensive understanding of energy quality and transformation processes.

The specific exergy and exergy values at each state are defined as [33,34,35]:

(33) $$e_{i}=(h-h_{0})-T_{0}(s-s_{0})$$

(34) $${\dot{E}}_{i}=\dot{m}e_{i}$$

Total exergy destruction rate of the S-CO_2_ cycle:

(35) $${\dot{E}}_{D}=\sum_{t=1}^{t=T}{\dot{E}}_{D,H}+{\dot{E}}_{D,t}+{\dot{E}}_{D,c}$$

where ${\dot{E}}_{D,H}$, ${\dot{E}}_{D,t}$ and ${\dot{E}}_{D,c}$ denote exergy destruction rate of heat exchangers, turbines and compressors.

Total exergy flowing into the S-CO_2_ cycle:

(36) $$\dot{E}=\dot{m}\left(e_{h,i}-e_{h,e}\right)$$

where $\dot{m}$ is the mass flow rate of the heat source medium; $e_{h,i}$ and $e_{h,e}$ are the specific exergies at the inlet and outlet of the heat source medium.

The exergy efficiency of the system is:

(37) $$\eta_{x}=\frac{\dot{W}}{\dot{E}}$$

### 3.3. Compactness Evaluation Model

The compactness of the power cycle system serves as a critical performance indicator, particularly in applications with spatial constraints such as aircraft carriers. If the system exhibits an excessively large volume, overly complex configuration, or exceeds the available installation space, its practical applicability will be significantly constrained. Given that heat exchangers constitute more than 70% of the total system volume, the heat exchange area per unit of output power is adopted as the objective function to quantitatively characterize the system’s compactness.

The heat exchange area per unit output power is the heat exchange area required by the system divided by the output power, that is:

(38) $$APR=\frac{\sum_{1}^{F}A_{f}}{\dot{W}}$$

where F denote the number of heat exchangers in the system; $A_{f}$ is the heat exchange area of the heat exchanger.

### 3.4. Exergy–Economic Model

Exergy–economic analysis integrates exergy analysis and economic analysis, offering insights that are unattainable through traditional thermodynamic analysis and economic evaluations. In exergy–economic analysis, the exergy cost balance equation and auxiliary equations are applied to each system component. The primary goal is to compute the cost of exergy flow per unit product and minimize the exergy cost to the greatest extent possible.

The exergy cost balance equation for system components involving heat transfer and work production is defined as:

(39) $$\sum {\dot{C}}_{e,k}+{\dot{C}}_{W,k}=\sum {\dot{C}}_{i,k}+{\dot{C}}_{Q,k}+{\dot{Z}}_{k}$$

In this equation, ${\dot{C}}_{W,k}$ and ${\dot{C}}_{Q,k}$ are the cost rates related to the work exergy flow and heat transfer exergy flow of the *k*th component, respectively; ${\dot{C}}_{i,k}$ and ${\dot{C}}_{e,k}$ denote the cost rates related to the inlet and outlet exergy flows of the kth component, respectively; ${\dot{Z}}_{k}$ is the cost rate related to the investment, operation, and maintenance costs of the kth system component.

Among them:

(40) $${\dot{C}}_{e,k}=c_{e,k}{\dot{E}}_{e,k}$$

(41) $${\dot{C}}_{i,k}=c_{i,k}{\dot{E}}_{i,k}$$

(42) $${\dot{C}}_{W,k}=c_{W,k}{\dot{E}}_{W,k}$$

(43) $${\dot{C}}_{Q,k}=c_{Q,k}{\dot{E}}_{Q,k}$$

In these equations, $c_{i,k}$ and $c_{e,k}$ are the unit exergy cost rates associated with the inlet and outlet fluids of the *k*th component, respectively. $c_{W,k}$ and $c_{Q,k}$ are the unit exergy cost rates related to the work and heat transfer of the *k*th component, respectively. ${\dot{E}}_{i,k}$ and ${\dot{E}}_{e,k}$ are the exergies of the inlet and outlet fluids of the *k*th component, respectively. ${\dot{E}}_{W,k}$ and ${\dot{E}}_{Q,k}$ are the exergies corresponding to the work and heat transfer of the *k*th component, respectively.

The cost rate associated with the investment cost of each component is given by:

(44) $${\dot{Z}}_{k}=Z_{k}\cdot CRF\cdot \varphi_{1}\cdot \varphi_{2}/(n\times 3600)$$

where $Z_{k}$ denote the investment cost of the *k*th component. As illustrated in Table 2 [36], the specific model can be derived accordingly. The currency employed and the base year are the US dollar and 2025, respectively. System costs consist of capital investment cost (CAPEX) and operating cost (OPEX) [37]. Capital investment incorporates both the direct and indirect costs of all major components within the nuclear reactor and power cycle. Operating costs embrace operation and maintenance expenses. $\varphi_{1}$ is the correction factor related to operation and maintenance, taken as 1.06, which accounts for system inspection and maintenance costs. $\varphi_{2}$ is a parameter that comprehensively takes into account various shipboard demand factors, including radiation shielding, redundancy and fault—tolerant design, sound insulation, shock and vibration resistance reinforcement, offshore maintenance, and system integration. The value of $\varphi_{2}$ is set to 1.2 [38,39,40]. n is the annual operating time.

The system investment recovery rate is expressed as:

(45) $$CRF=\frac{i{\left(1+i\right)}^{n}}{{\left(1+i\right)}^{n}-1}$$

where i represents the real discount rate 0.05, and n represents the number of years of system operation, 7000 h [41].

The cost rates of unit fuel exergy and product exergy are as follows:

(46) $$c_{F,k}=\frac{{\dot{C}}_{F,k}}{{\dot{E}}_{F,k}}$$

(47) $$c_{P,k}=\frac{{\dot{C}}_{P,k}}{{\dot{E}}_{P,k}}$$

where ${\dot{C}}_{F,k}$ and ${\dot{C}}_{P,k}$ are the cost rates of the fuel exergy and product exergy of the *k*th component, respectively. ${\dot{E}}_{F,k}$ and ${\dot{E}}_{P,k}$ are the fuel exergy and product exergy of the *k*th component, respectively.

The cost rate related to exergy loss is defined as:

(48) $${\dot{C}}_{D,k}=c_{F,k}{\dot{E}}_{D,k}$$

where ${\dot{E}}_{D,k}$ is the exergy loss of the *k*th component.

The exergy efficiency of each component is expressed as:

(49) $$\eta_{ex,k}=\frac{{\dot{E}}_{P,k}}{{\dot{E}}_{in,k}}$$

where ${\dot{E}}_{P,k}$ and ${\dot{E}}_{in,k}$ represent the output and input exergies of the *k*th component, respectively.

The exergy-economic factor, which represents the significance of the investment cost rate of a component relative to the exergy-loss cost rate, is expressed as:

(50) $$f_{k}=\frac{{\dot{Z}}_{k}}{{\dot{Z}}_{k}+{\dot{C}}_{D,k}}$$

The objective function of exergy economy, which is the cost rate per unit output power, is expressed as:

(51) $$\mathrm{LEC}=\frac{\sum_{1}^{n_{k}}{\dot{Z}}_{k}+{\dot{C}}_{fuel}}{\sum_{1}^{n_{p}}{\dot{E}}_{P,i}}$$

where $n_{k}$ and $n_{P}$ represent the number of components and product flows in the system. ${\dot{C}}_{fuel}$ represents the cost rate of fuel exergy. ${\dot{E}}_{P,i}$ refers to the exergy of the *i*th product flow.

### 3.5. Model Validation

To validate the accuracy of the supercritical carbon dioxide system model employed in this study, a comparison was made with relevant published data. Lee et al. [23] investigated a supercritical carbon dioxide Brayton cycle power generation system utilizing a reactor. Table 3 presents a comparative analysis of the performance results obtained from the present model and those reported in Ref. [23]. Through this comparison, it was found that when the input conditions were identical, the deviations of key performance parameters between the present model and the data in Ref. [23] were all below 5%. This high degree of consistency confirms the validity and reliability of the model proposed in this study. In order to validate the performance of the components, a comparison was made between the calculations of the turbine and the heat exchanger. The comparison results are presented in Table 4. As can be observed from Table 4, the error is less than 5%. This thereby validates the correctness of the calculation method employed in this study.

## 4. Results and Discussion

### 4.1. Mechanism Analysis of Compactness in Supercritical Carbon Dioxide Cycle

The high compactness of the S-CO_2_ system is primarily attributed to the smaller size of its turbine compared to those in other cycles. The radial size of the power machinery is closely tied to the volume flow rate. To evaluate this compactness, the specific power volume flow rate of the turbine is used as a key metric, as it directly correlates with the diameter of the turbine’s outlet impeller.

**Figure 6:**
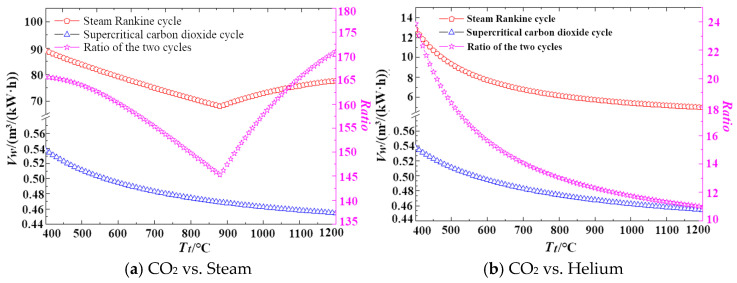
Comparison chart of specific power volume flow of different cycle types.

Figure 6 illustrates the specific power volumetric flow rates for various cycle types. As shown in Figure 6a, the specific power volumetric flow rate (*V_w_*) of the steam Rankine cycle exceeds that of the S-CO_2_ cycle by more than two orders of magnitude. Similarly, Figure 6b shows that the helium cycle’s specific power volumetric flow rate is over an order of magnitude higher than that of the S-CO_2_ cycle.

**Figure 7:**
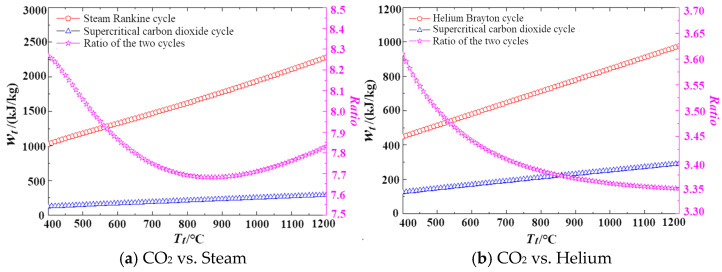
Comparison of expansion specific work of different cycle types.

**Figure 8:**
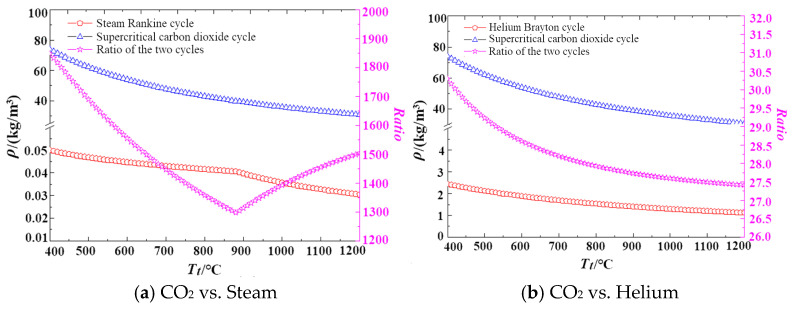
Density comparison chart of different cycle types.

The specific power volumetric flow rate of a turbine is defined as the ratio of specific work of expansion to density. Figure 7 and Figure 8 analyze the factors contributing to these differences. Figure 7 compares the specific work of expansion across the three cycles, revealing that their values are of the same order of magnitude with no significant variations. In contrast, Figure 8 compares the densities at the turbine outlet, showing that the density of the supercritical carbon dioxide cycle is more than 1000 times higher than that of the steam Rankine cycle, and over 20 times greater than that of the helium cycle. This indicates that the primary factor influencing the differences in specific power volumetric flow rate is the variation in density.

The high density during the expansion process is a key advantage of the compact S-CO_2_ cycle. The molecular structure of supercritical carbon dioxide exhibits slightly weaker intermolecular attraction than gaseous CO_2_, yet maintains a molecular distance comparable to liquid CO_2_. This results in a degree of molecular aggregation similar to that of liquid carbon dioxide, giving supercritical carbon dioxide its characteristic high density.

As shown in Figure 6a, the specific power volumetric flow rate of the steam Rankine cycle initially decreases and then increases with rising temperature, exhibiting a trend similar to that of the S-CO_2_ cycle. This behavior can be explained using Figure 8a: in the steam Rankine cycle, with a constant evaporation dew point temperature, the rise in the turbine inlet temperature increases the degree of superheat. Consequently, the dryness at the turbine outlet also increases. When the turbine outlet temperature exceeds 870 °C, the dryness exceeds unity, transitioning the working medium into a fully gaseous state. This transition causes the density to decrease more sharply as the turbine inlet temperature rises. Initially, the density decreases gradually, but beyond a certain point, it declines rapidly, resulting in the observed trend in specific power volumetric flow rate. This analysis highlights that very high turbine inlet temperatures negatively impact the compactness of the steam Rankine cycle.

**Figure 9:**
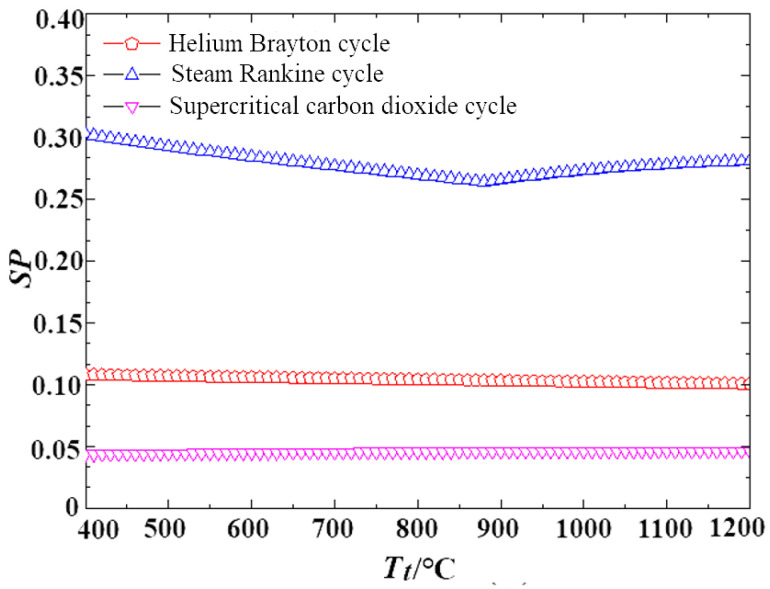
Comparison of size parameters of different cycles.

Furthermore, the size parameter of the expander serves as an effective indicator for quantifying the actual dimensions of the expander, as expressed in Equation (52). Figure 9 compares the size parameters across the three cycles, demonstrating that the S-CO_2_ cycle has the smallest size parameter among them. This highlights the superior compactness of the S-CO_2_ cycle compared to the steam Rankine and helium cycles.

(52) $$SP=\frac{\sqrt{V_{\mathrm{out}}}}{\sqrt[{4}]{\Delta H_{is}}}=\frac{\sqrt{m_{out}/\rho_{out}}}{\sqrt[{4}]{\Delta H_{is}}}$$

where *m_out_* and *ρ_out_* represent the mass flow rate and density at the expander outlet, respectively; Δ*H_is_* is the isentropic enthalpy drop of the expander.

### 4.2. Parametric Studies

**Figure 10:**
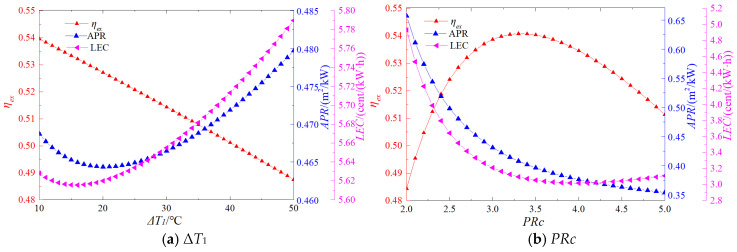

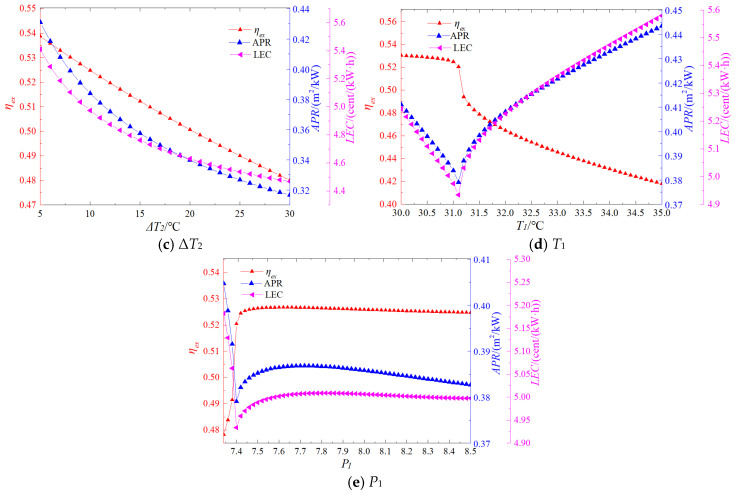
Effect of operating parameters on system performance (**a**) Δ*T*_1_, (**b**) *PRc*, (**c**) Δ*T*_2_, (**d**) *T*_1_, (**e**) *P*_1_.

In this section, a systematic investigation is conducted to examine the influence of system operating parameters on key performance indicators. The selected operating parameters include the temperature difference between the heat source and supercritical carbon dioxide (Δ*T*_1_), the pressure ratio of the S-CO_2_ cycle (PRc), the narrow-point temperature difference in the regenerator (Δ*T*_2_), the compressor inlet temperature (T_1_), and the compressor inlet pressure (P_1_). The chosen performance indicators are critical to system design and practical application. These include exergy efficiency, levelized energy cost (LEC), and heat transfer area per unit power output (APR). Figure 10 illustrates the observed influence patterns of these decision variables on the corresponding performance indicators.

As illustrated in Figure 10a, the exergy efficiency decreases with an increase in Δ*T*_1_. This trend can be attributed to the reduction in turbine inlet temperature under larger temperature differences, which lowers the heat absorption temperature and consequently diminishes system efficiency. In contrast, both LEC and APR first decrease and then increase as Δ*T*_1_ increases, reaching their minimum values at 16 °C and 20 °C, respectively. This behavior arises because, although a higher Δ*T*_1_ reduces output power, it also reduces the required heat exchange area, thereby lowering costs. Initially, the decline in power output dominates; however, beyond a certain threshold, the reduction in heat exchange area becomes the dominant factor, leading to the observed trends in LEC and APR.

Figure 10b demonstrates that exergy efficiency initially rises with increasing *PRc*, peaks at approximately 3.3, and subsequently declines. Meanwhile, LEC continues to decrease and reaches its optimal value at a pressure ratio of 4. These findings indicate that, for the supercritical CO_2_ regenerative cycle, a higher-pressure ratio does not necessarily yield superior overall performance.

As shown in Figure 10c, all three performance indicators—exergy efficiency, LEC, and APR—decrease with an increase in Δ*T*_2_. A larger regenerator narrow-point temperature difference results in reduced system output power and increased heat exchange area. Consequently, while such an increase negatively affects thermodynamic performance, it offers economic benefits by reducing equipment size and associated costs.

Figure 10d illustrates that exergy efficiency gradually declines and then sharply drops as the compressor inlet temperature increases. Once the temperature deviates from the critical point, the rate of decline slows. Similarly, LEC and APR exhibit a non-linear trend, decreasing initially and then increasing with rising compressor inlet temperature, reaching their lowest values at 31.1 °C. As depicted in Figure 10e, exergy efficiency increases rapidly at first and then more gradually with rising compressor inlet pressure. Beyond the critical pressure, the rate of improvement diminishes. Likewise, LEC and APR follow an initial decreasing and subsequent increasing trend with respect to compressor inlet pressure. Therefore, when the compressor inlet conditions approach the critical point, both thermodynamic and economic performance reach optimal levels. This phenomenon is primarily due to the relatively low compression work required when CO_2_ is compressed near its critical state.

### 4.3. Multi-Objective Optimization Study

By parameter analysis, it has been determined that the optimal value of each objective function corresponds to distinct system operating parameters. This indicates that no single set of parameters can simultaneously optimize all performance metrics, as mutual constraints exist among them. To address this, the system’s key operational parameters are treated as decision variables, and a multi-objective optimization approach is applied to achieve optimal performance.

The parameters significantly influencing system performance are selected as decision variables: the temperature difference between the heat source and S-CO_2_ (Δ*T*_1_), the S-CO_2_ cycle pressure ratio (*PRc*), the narrow-point temperature difference in the regenerator (Δ*T*_2_), the compressor inlet temperature (*T*_1_), and the compressor inlet pressure (*P*_1_). Their upper and lower bounds are provided in Table 5.

Given the large number of decision variables and the high precision required for optimization, the genetic algorithm is employed for multi-objective optimization. Initially, the genetic algorithm generates the Pareto frontier, which represents the set of optimal solutions, with each point being a feasible solution. Subsequently, the Technique for Order Preference by Similarity to an Ideal Solution (TOPSIS) decision-making method is used to identify the ultimate optimal solution, yielding the system’s final operating parameters.

#### 4.3.1. Dual-Objective Function Optimization

Dual-objective function optimization involves the simultaneous optimization of two objective functions to achieve an optimal balance. In this system, three key performance indicators—exergy efficiency (*η_x_*), levelized energy cost (LEC), and heat transfer area per unit power output (APR)—are available. Based on the operational requirements of an aircraft carrier, where volume and weight constraints are critical, exergy efficiency and APR are chosen as the objective functions for optimization in this section.

**Figure 11:**
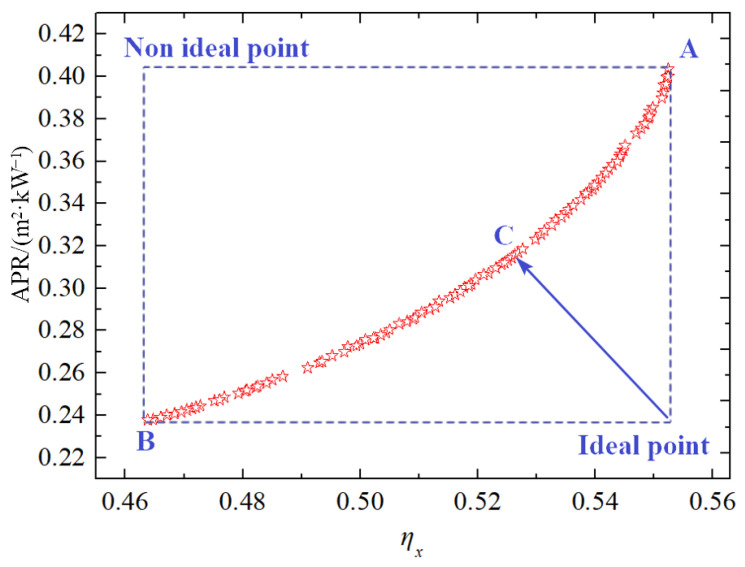
Pareto frontier of total product unit cost with exergy efficiency.

Figure 11 illustrates the Pareto frontier solutions of the system. At point A, the exergy efficiency is 55.2%, and the APR is 0.404 m^2^ kW^−1^, representing the best thermodynamic performance but the poorest compactness. Conversely, at point B, the exergy efficiency is 46.3%, and the APR is 0.238 m^2^ kW^−1^, signifying the best compactness but the poorest thermodynamic performance. Notably, no point on the Pareto frontier achieves both maximum exergy efficiency and minimum APR simultaneously. The points corresponding to these extremes are referred to as ideal points.

Using the TOPSIS decision-making approach, point P, which is closest to the ideal points, is identified as the optimal solution on the Pareto frontier. At point P, the exergy efficiency is 52.8%, and the APR is 0.312 m^2^ kW^−1^. The corresponding decision variables are: Δ*T*_1_ = 27 °C, *PRc* = 2.7, Δ*T*_2_ = 12 °C, *T*_1_ = 31.5 °C, and *P*_1_ = 7.42 MPa.

#### 4.3.2. Three Objective Function Optimization

**Figure 12:**
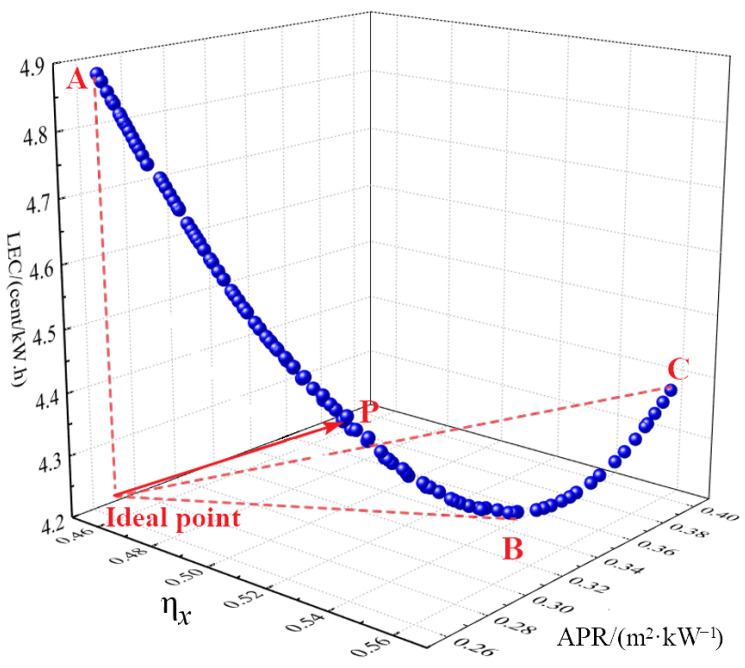
Three-dimensional optimization results of the three objective functions of the system.

When it becomes necessary to adopt additional dimensions to evaluate system performance in practical applications, exergy efficiency (*η*_x_), LEC, and APR can be simultaneously used as objective functions for the system’s three-objective optimization. This approach enables a comprehensive assessment of thermodynamic performance, spatial compactness, and economic performance within the combined cycle. The Pareto frontier solutions obtained from this optimization are shown in Figure 12.

From Figure 12, it can be observed that point C achieves the maximum exergy efficiency of 56%, point B attains the minimum LEC of 4.25 cent/(kW·h), and point A exhibits the minimum APR of 0.257 m^2^ kW^−1^. These results indicate that point C represents the best thermodynamic performance, point B the best economic performance, and point A the most favorable spatial performance. However, no single point simultaneously optimizes all three performance metrics. Consequently, similar to the two-objective optimization approach, a decision-making process is employed using a hypothetical ideal point, defined by maximum exergy efficiency, minimum APR, and minimum LEC. The optimal point is identified as the solution on the Pareto frontier that is closest to this ideal point, designated as point P.

**Figure 13:**
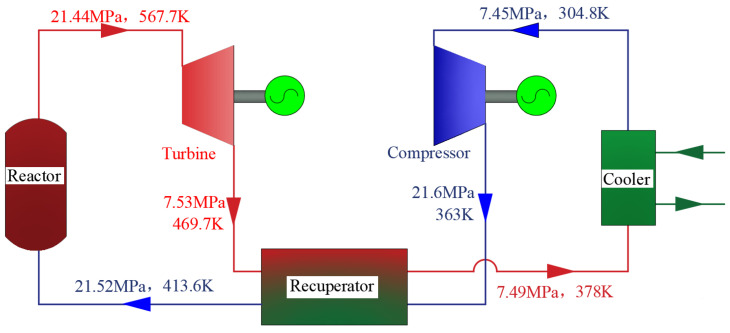
Diagram of the Optimized System Operating Parameters.

At point P, the corresponding optimal exergy efficiency, APR, and LEC are 51%, 0.272 m^2^ kW^−1^, and 4.5 cents/(kW·h), respectively. The value of LEC is 10% lower than the nuclear power cost reported in the literature [36]. The decision variables for this optimal solution are: Δ*T*_1_ = 24 °C, *PRc* = 2.8, Δ*T*_2_ = 14 °C, *T*_1_ = 31.6 °C, and *P*_1_ = 7.45 MPa. The results of the final system operation parameters are presented in Figure 13. The turbine is configured with a single-cylinder double-flow structure. The detailed structural parameters are presented in Table 6.

## 5. Summary and Conclusions

This paper proposes an innovative integrated system for nuclear-powered supercritical carbon dioxide (S-CO_2_) applications in aircraft carriers. The system is divided into two modules: the nuclear reactor module and the S-CO_2_ power module. The S-CO_2_ power cycle adopts a regenerative cycle, utilizing carbon dioxide as the working fluid, which demonstrates excellent environmental friendliness. Comprehensive analyses, including thermodynamic performance, economic evaluation, and spatial compactness assessments, were conducted based on application requirements. The key conclusions are as follows:

(1) The specific power volume flow rate of the steam Rankine cycle is over 100 times greater than that of the S-CO_2_ cycle, and that of the helium cycle is more than 10 times greater. The primary reason for the superior compactness of the S-CO_2_ cycle is its high density during the expansion process.
(2) Optimal thermodynamic and economic performance is achieved when the compressor inlet conditions are near the critical point. This is due to the low power consumption required to compress carbon dioxide in this state.
(3) The system was subjected to a dual-objective optimization with exergy efficiency and APR as the objective functions. The optimization outcomes demonstrated that the optimal exergy efficiency and APR were 52.8% and 0.312 m^2^ kw^−1^, respectively. The corresponding decision variables were as follows: Δ*T*_1_ was 27 °C, *PRc* was 2.7, Δ*T*_2_ was 12 °C, *T*_1_ was 31.5 °C, and *P*_1_ was 7.42 MPa.
(4) The system was conducted with triple-objective optimization with exergy efficiency, APR, and LEC as the objective functions. The optimization results reveal that the optimal exergy efficiency, APR, and LEC are 51%, 0.272 m^2^ kW^−1^, and 4.5 cent/(kW·h), respectively. The corresponding decision variables are: Δ*T*_1_ is 24 °C, *PRc* is 2.8, Δ*T*_2_ is 14 °C, *T*_1_ is 31.6 °C, and *P*_1_ is 7.45 MPa. Therefore, the supercritical carbon dioxide power module exhibits high compactness and is feasible for application in the nuclear power of aircraft carriers.

## Figures and Tables

**Table 1:** Parameters of Nuclear Reactors [25].

Parameters	Value
Rated thermal power of the reactor/MW	385
Fuel assembly type	CF3S17 × 17 square arrangement
Length of fuel active section/m	2.15
Quantity of fuel assemblies	57
Fuel reload period/year	50
Operating pressure of the reactor coolant system/MPa	15
Number of control rod driving mechanisms	21
The quantity of main cooling pumps	4
The inner diameter of the reactor pressure vessel/m	3.36

**Table 2:** The investment cost of the components [36].

Components	Investment Cost
Reactor	$Z_{reac}=c_{reac}.Q_{reac}$
Turbine	$Z_{tur}=479.34m(\frac{1}{0.93-\eta_{tur}})\ln(PR_{tur})\cdot (1+\exp(0.036T_{1}-54.4))$
Compressor	$Z_{com}=71.1m(\frac{1}{0.92-\eta_{com}})\ln(PR_{com})PR_{com}$
Heat exchangers	$Z_{H}=2681\cdot A_{k}^{0.59}$

**Table 3:** Model verification.

Operation and Performance Parameters		Reference	This Model
Input parameters	MC inlet pressure/MPa	8.5	8.5
	Pressure ratio	2.9	2.9
	Flow split ratio	0.7	0.7
	Thermal power/MWth	60.97	60.97
Output parameters	Cycle Net power/MWe	29.13	30.02
	Cycle efficiency/%	47.78	47.93

**Table 4:** Components verification.

Component	Turbine Verification [42]			Heat Exchanger Validation [43]		
	Parameters	Reference	This Model	Parameters	Reference	This Model
Input parameters	Turbine inlet temperature/K	673.15	673.15	Inlet temperature/°C	76.2	76.2
	Turbine inlet temperature/MPa	19.31	19.31	Outlet temperature/°C	34.4	34.4
	Turbine exit pressure/MPa	7.63	7.63	Inlet pressure/MPa	8.22	8.22
	Mass flow rate/(kg/s)	12.74	12.74	Flow rate/(kg/s)	18.06	18.06
Output parameters	Power output of turbine/MW	1.16	1.20	Heat transfer rate per unit volume/(MW/m^3^)	16.54	17.12
	Turbine efficiency/%	85.36	87.62	Pressure drop/kPa	4.20	4.05

**Table 5:** Upper and lower boundaries of the decision variable.

Decision Variables	Lower Bound	Upper Bound
Δ*T*_1_/°C	10	50
*PRc*	2	4.2
Δ*T*_2_/°C	5	30
*T*_1_/°C	31	35
*P*_1_/MPa	7.4	9

**Table 6:** Turbine geometric parameters.

Parameters	Unit	Value
Number of Stages	-	2 × 6
Rotational Speed	r/min	3000
Blade Height Range	mm	68–107
Root Diameter	mm	650
Configuration	-	Single—cylinder Double—flow
Efficiency	%	85
Shaft Power	MW	128

## Data Availability

Data is contained within the article.
